# A computational framework for modeling fluid and solid stresses in porous tissues with application to the trabeculated embryonic heart

**DOI:** 10.1371/journal.pcbi.1014896

**Published:** 2026-10-23

**Authors:** Christine Miller Buffinton, James W. Baish

**Affiliations:** 1 Department of Mechanical Engineering, Bucknell University, Lewisburg, Pennsylvania, United States of America; 2 Department of Biomedical Engineering, Bucknell University, Lewisburg, Pennsylvania, United States of America; Islamic Azad University Arsanjan Branch, IRAN, ISLAMIC REPUBLIC OF

## Abstract

This study provides a framework for efficient solutions of computational fluid-solid interaction models of porous tissue, with a focus on blood flow through the developing heart wall in the critical chamber and valve formation stages, when trabeculation fills much of the ventricles. This porous, trabeculated tissue is the site of complex mechanotransduction processes, but accurately estimating solid and fluid stresses within such intricate geometries is difficult. We addressed this with three levels of modeling and verification. Images of E12.5 mouse ventricles were first obtained through confocal microscopy and 3D reconstruction. These guided creation of explicit pore-scale finite element simulations with fluid-solid interaction. Idealized and image-derived unit cells were analyzed to determine hydraulic permeability. Next, a multi-pore representation of the ventricular wall was solved with full fluid and solid equations. A continuum model based on Brinkman equations then replaced the explicit pore structure, and sensitivity to permeability was evaluated in terms of fluid flow in the open channel and porous region. Permeabilities obtained from unit-cell simulations followed trends from Kozeny-Carman solid-pore and fiber theory for the rigid case, while simulations for hyperelastic unit-cell material quantified the effects of tissue elasticity. Comparison of continuum and explicit model results showed the Brinkman equation, which incorporates the diffusion of shear stress from flow in the free fluid into the porous media, but not explicitly inertial effects, could significantly improve the accuracy of continuum models compared to Darcy’s law. A 3D model of blood flow through the ventricles of the developing mouse heart demonstrated the application of the technique to more realistic geometry, showed locations of peak solid and shear stress, and produced physiologically reasonable flow. Furthermore, we developed methodologies to estimate local wall shear and solid stress from continuum-based calculations. This approach can enable efficient, whole-organ parametric analyses of fluid flow, circumventing the need for computationally expensive explicit pore modeling.

## Author summary

Many biological tissues are porous, allowing the passage of fluids such as blood, lymph, and interstitial fluid across the tissue with varying levels of resistance. One important example is the porous, trabecular tissue filling the ventricles of the developing heart before formation of coronary circulation. The response of this trabecular region to mechanical load suggests it is an important sensor and actuator for mechanotransduction. Although computational modeling of fluid flow interacting with complex, porous, elastic tissue can help elucidate details of mechanotransduction, the analysis is computationally intensive, propagates uncertainties from image analysis and meshing, and does not easily allow parameter studies. We addressed this challenge with anatomical studies of the 12.5-day mouse heart, followed by three levels of modeling, integrating single-pore simulations, layered models of solid and porous regions with impinging fluid, and continuum representations of the porous region. The continuum model was efficient and agreed well with explicit pore representations. A ventricular model of 12.5-day mouse demonstrated application to more realistic geometry. We also developed methods to estimate local stress from continuum-based calculations. This approach enables efficient, whole-organ parametric analyses of fluid flow, circumventing the need for computationally expensive explicit pore modeling.

## 1 Introduction

This study provides a framework for efficient solutions of computational fluid dynamic (CFD) models of porous tissue to aid in assessing the effects of fluid loading on the mechanical environment that influences mechanotransduction processes. We use the developing heart in the chamber and valve formation stages to demonstrate the application of this framework. In developing cardiovascular tissue, hemodynamic loading creates solid tissue stress and wall shear stress (WSS), which interact with local mechanical properties to create responses at the genetic, molecular, and cellular levels. Developmentally appropriate stimuli are necessary for normal development, while interventions altering pressure and flow at early stages have been shown across organisms to influence subsequent morphogenesis and ultimate cardiac structure and function [1–6].

Models at these cardiac developmental stages are complicated by the presence of myocardial trabeculation, a porous network of myocardial fibers covered in endocardium filling much of the ventricular chambers in common experimental animals such as mouse and chick, and in human, and allowing perfusion of the wall before the appearance of coronary arteries. During development, paired endocardial heart tubes fuse to form a single beating heart tube [7]. The tube then elongates and loops before beginning chamber and valve formation (Fig 1). Trabeculation begins at the end of this cardiac looping phase, as myocardial evaginations project from the endocardium toward the jelly, eventually reaching the smooth surface of the myocardium [8]. Trabeculae appear at Hamburger-Hamilton (HH) stage HH15 or embryonic day post-fertilization (E) E2.3 in chick, where they are visible to HH25 (E4.5) [8,9], and the end of the 4^th^ gestational week in human. In mouse, trabeculae form a meshlike network by E10.5 [10]. As ventricular septation proceeds (E10.5–E14.5, Fig 2), the trabecular mesh becomes more densely packed and complex, later coalescing into the compact wall.

**Fig 1:**
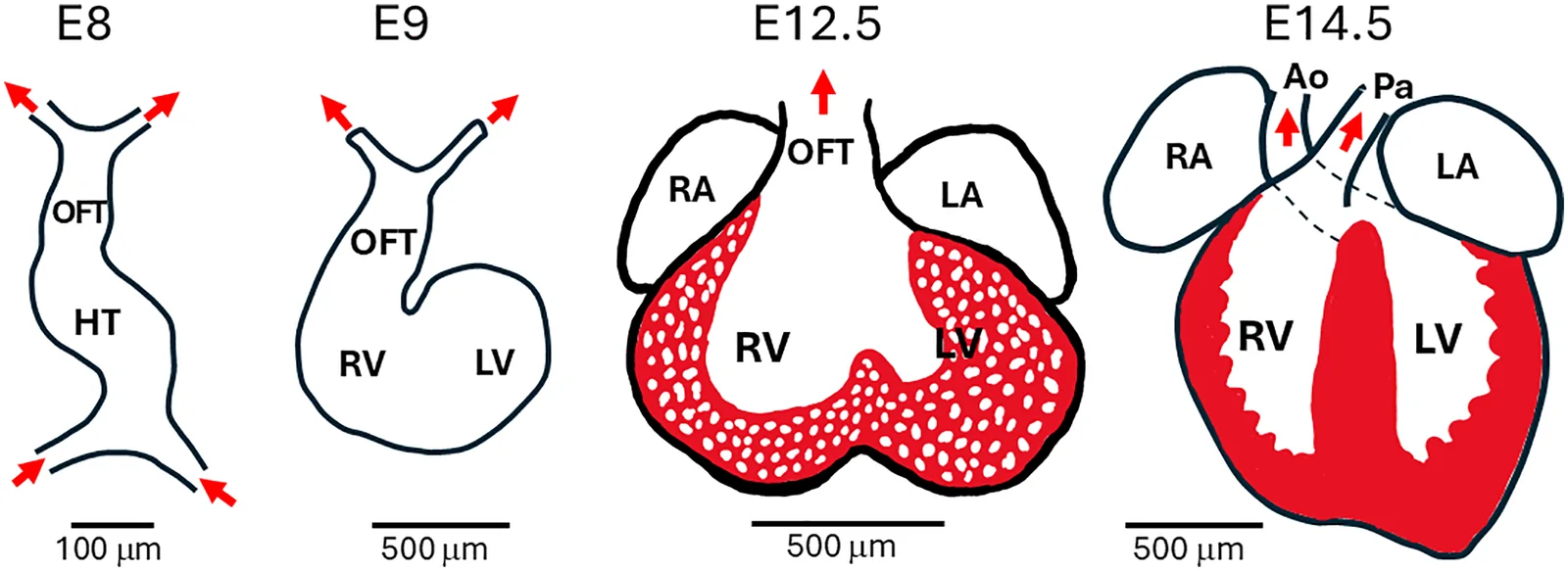
Schematic of mouse heart development from tubular to four-chambered. Days of gestation are labeled “E” and arrows show the direction of blood flow. OFT: outflow tract, HT: heart tube, RV: right ventricle, LV: left ventricle, RA: right atrium, LA: left atrium, Ao: aorta, PA: pulmonary artery. The red material lining the ventricles at E12.5 represents trabeculated myocardium, while the more solid red wall at E14.5 shows coalescence into the compact wall. Dimensions from comparison with [11].

**Fig 2:**
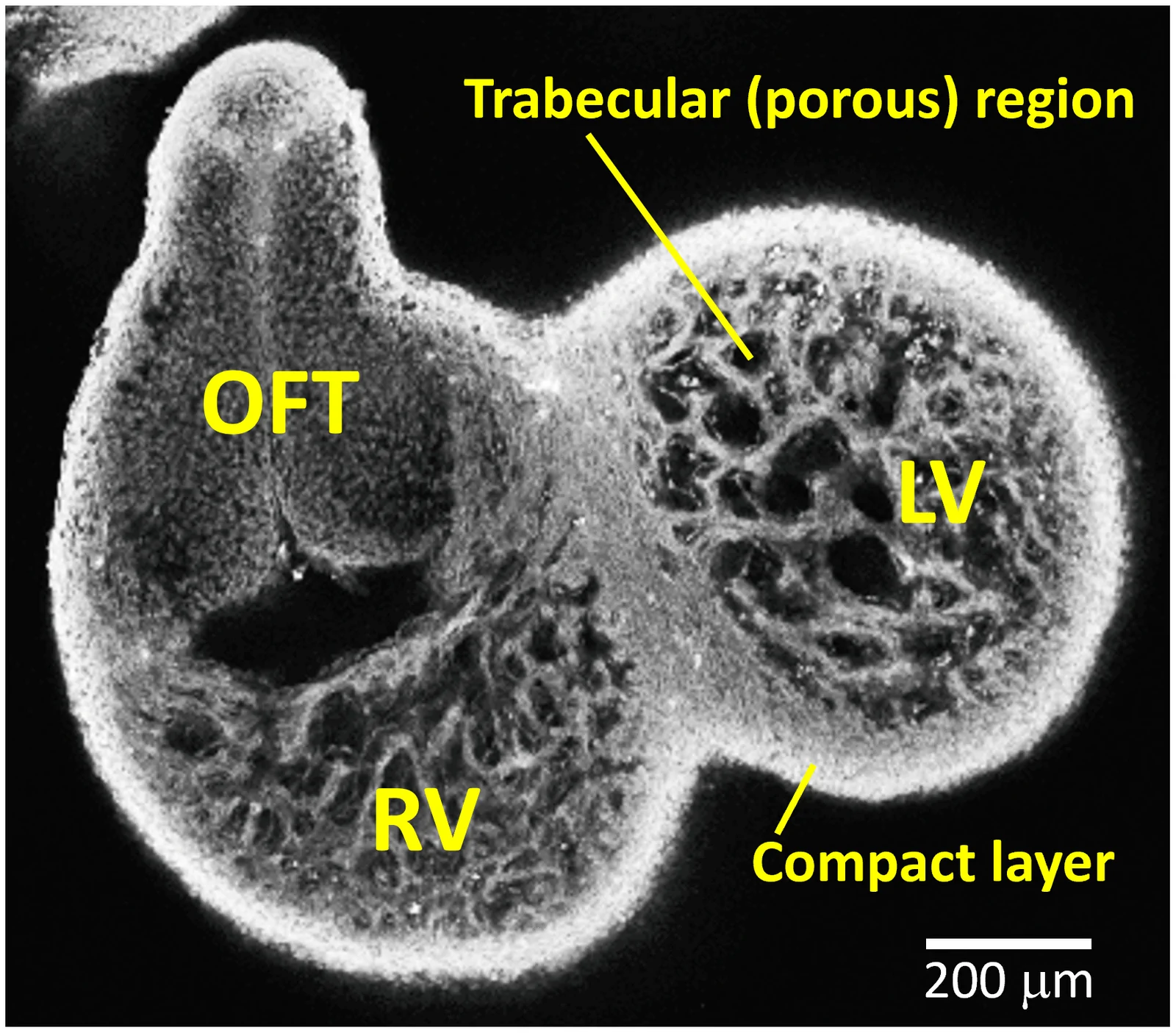
Section through E12.5 mouse heart. This image from confocal microscopy illustrates trabeculation in the ventricular chambers. OFT: outflow tract, RV: right ventricle, LV: left ventricle.

The prominence of this porous layer suggests a substantial impact on flow. In the developing heart, observation that shear stresses are high at the trabeculae [12] and their orientation changes with abnormal ventricular pressure [2] suggests a role in mechanotransduction. The complete geometry of the trabeculated ventricles can be determined by imaging and 3D reconstruction [13], but explicit CFD analysis with fluid-structure interaction (FSI) on such models is computationally intensive, introduces and propagates uncertainties from image analysis and meshing, and perhaps most importantly, does not easily allow parameter sensitivity studies.

This study uses the embryonic mouse heart, a common animal model for studying human congenital heart disease [14,15], to develop and verify the method. Pore spaces in the trabeculated region are numerous and small, but not to the extent that a continuum model averaging over numerous microstructures can be naively applied. Typically, continuum models of porous tissues are most appropriate when the scale of the pores is at least two orders of magnitude smaller than the macroscopic scale of the tissue [16]. In the developing mouse heart, a trabecular pore may be 50 µm long, while the compact wall is only a few hundred microns thick, raising the question of how a continuum model can be used effectively. This study answers that question with three levels of modeling and verification.

The first model level (Fig 3a) was a single unit cell, with fluid flow through the pore modeled with the full Navier-Stokes equations and the solid structure governed by neo-Hookean hyperelastic material properties. The aim was to relate through-flow and pressure drop across a representative volume of the heart wall to yield a specific hydraulic permeability. We employed models with idealized unit cell geometry as well as image-based models from scans similar to Fig 2. The second level was a 3D, explicit, multi-pore model of an idealized section of the heart wall solved with the full fluid and solid equations (Fig 3b). The aim here was to examine how free-flowing fluid impinging on the porous wall from the open central cavity of a ventricle passes through the trabecular spaces. Finally, a third model with the same configuration as the 3D model replaced the explicit representation of the unit cell structure with a continuum model employing an effective permeability (Fig 3c). This continuum model is intended to be suitable for use in a whole-organ model where the explicit model would be computationally impractical. The utility of the continuum representation was demonstrated in an idealized 3D model of the left (LV) and right (RV) ventricles of the E12.5 mouse heart.

**Fig 3:**
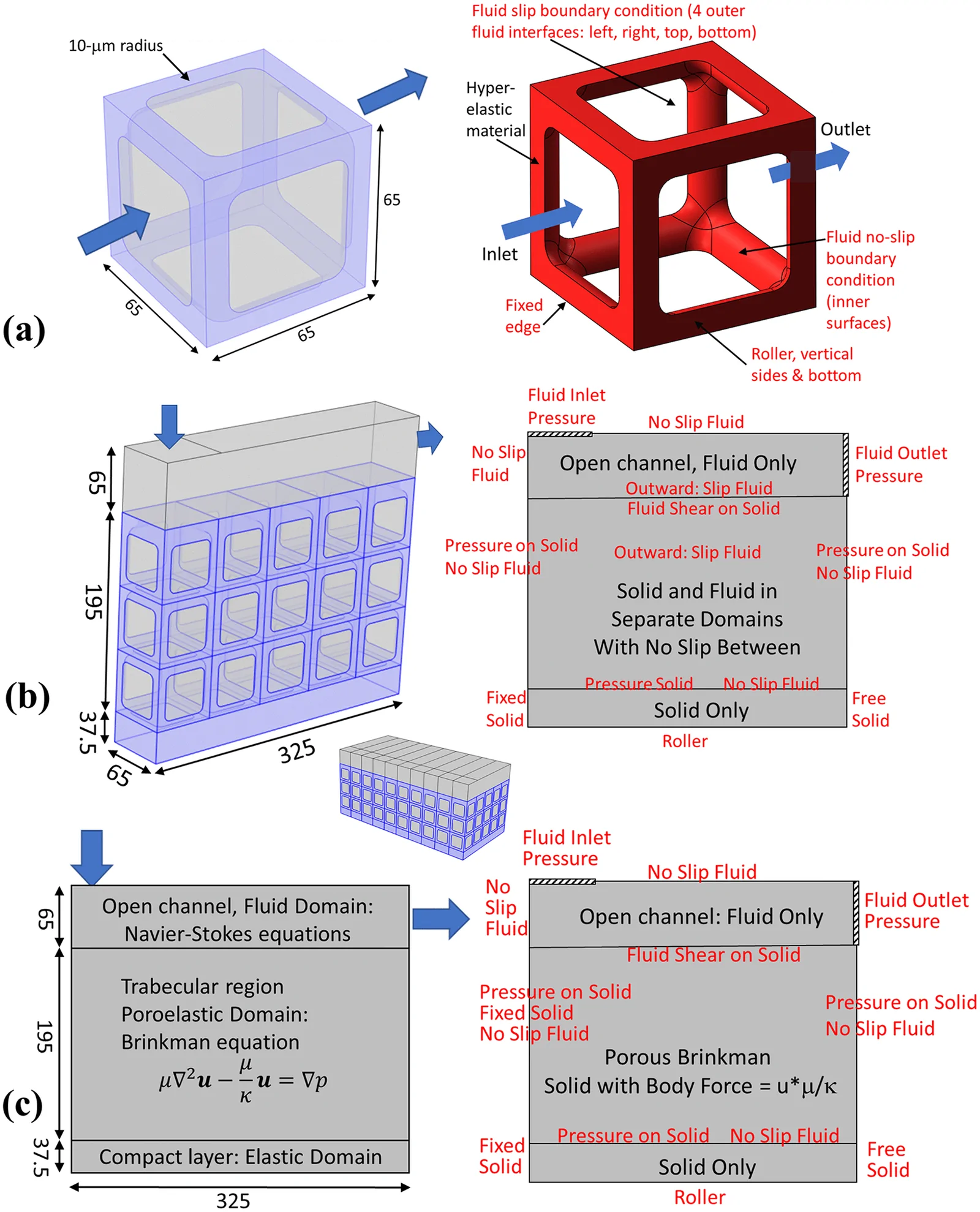
Three levels of idealized models. Images on the left show geometry with dimensions in μm and arrows indicating direction of fluid flow; images on the right show boundary conditions. (a) Unit cell model shown with nominal dimensions with solid domains in blue and fluid domains in gray. (b) 3D explicit model, with solid domains in blue and fluid domains in gray. (c) 2D continuum model.

The ultimate aim of this research is to relate results obtainable from the continuum model back to the shear and bending stresses expected in porous structures, these stresses being most relevant to the mechanobiological processes.

## 2 Methods

### 2.1 Ethics statement

Animal protocols for obtaining images of E12.5 mouse hearts were carried out in strict accordance with the recommendations in the Guide for the Care and Use of Laboratory Animals of the National Institutes of Health. The protocol was approved by the Weis Center for Research/Geisinger Clinic Institutional Animal Care and Use Committee (IACUC), protocol number 203–17. Maternal euthanasia followed the 2013 American Veterinary Medical Association (AVMA) Guidelines on Euthanasia: Recommended and Acceptable with Conditions.

### 2.2 Governing equations

The trabecular layer of the developing heart is a saturated porous medium. Two methods can be used to mathematically model fluid flow in a porous medium. In the first, the governing equations, usually the Navier-Stokes equations, are solved in individual pores using the exact geometry. An advantage to this method is geometric specificity, which can also be a disadvantage for complicated domains, as the solid geometry must be accurately represented. Alternatively, a continuum approach represents the porous medium by bulk, average parameters, generally valid when the average size of the pores is much less than some characteristic distance, usually the distance over which macroscopic changes in physical quantities such as pressure and velocity are significant. The medium is characterized by a liquid phase with volume fraction ɛ, the porosity, and a solid phase with volume fraction (1-ɛ).

The simplest continuum representation of flow in porous media is Darcy’s law, given by


$$\begin{array}{c} -\frac{\mu }{k}u\;=\nabla p\;, \end{array}$$ (1)

where **u** (m/s) is the fluid velocity, *κ* (m^2^) is specific hydraulic permeability of the porous material, μ the dynamic viscosity of the fluid (Pa-s), and ∇p the pressure gradient (Pa/m). Darcy’s law is most suitable away from boundaries with free-flowing fluid such as exist between the trabeculated tissue and the blood in the open ventricle chamber. The Darcy approximation is also best when *κ* is very low, such as in fluid percolation through interstitial spaces, where κ can be as low as 10^-17^ m^2^ and Reynold’s numbers in the pores less than 10^-10^ [17]. In the developing mouse heart, porosity of the trabecular region can be more than 50% [13]. The large pore size suggests that shear stress from the free-fluid domain can impose flow in the porous medium. The Brinkman equations for porous media flow may then be more accurate, given by


$$\begin{array}{c} \mu \nabla^{\text{2}}u-\frac{\mu }{\kappa }u\;=\nabla p\;. \end{array}$$ (2)

Darcy’s law neglects the first term of (2), which should be included when $\kappa^{\frac{\text{1}}{\text{2}}}=O(L),$ where L is the characteristic length over which macroscopic changes in the velocity must be considered, here the thickness of the trabeculated tissue [16].

### 2.3 Idealized unit-cell computational model for estimation of permeability

Solution of Eq. (2) requires specification of viscosity and permeability. Using the E12.5 mouse heart as our animal inspiration, we estimated permeability by designing an idealized geometric model of a single pore structure from the trabecular region of the heart, modeling flow through this unit cell with a fully coupled CFD-FSI finite element approach, and extracting permeability from Eq. (1), which could be applied due to the low Reynolds numbers and lack of immediate interest in fluid-porous boundaries.

The unit-cell model was inspired by literature on ceramic and metallic foams, where typical geometries are polyhedral and pressure drop across the material is low due to high porosity [18,19]. The unit cell geometry was defined by cylindrical struts centered on the 12 edges of a cube and truncated at the cube edges to represent interfaces with identical, adjoining cells (Fig 3a). Unit cell dimensions were obtained from images of E12.5 mouse hearts. After maternal euthanasia, ED12.5 mouse embryos were removed into 1x PBS on ice. Hearts were dissected from the embryos, preserving the atria, ventricles, and outflow tract. Hearts were then arrested in diastole with verapamil, treated for RBC lysis, flushed, fixed in paraformaldehyde, dehydrated in EtOH, cleared in BABB, and imaged by confocal microscope; see [13] for details. Model dimensions were the average of the side-to-side extent of the solid structures and width of myocardial struts taken from 10 measurements on each of 10 sections at mid-level from 10 hearts in two groups, RV and LV. The geometry was created in SolidWorks from the average measurements of 65-μm cube side and 10-μm strut radius; this will be referred to as the nominal or 65–10 unit cell. We examined the effects of varying unit cell sizes within an organ by also analyzing cubes of 0.5, 1.5, and 2.0 times nominal length and struts of the same radius relative to cube side plus 20% smaller, 20% larger, and 50% larger, and one additional 10% smaller strut for the 130-μm cube. Rectangular cuboid unit cells with 1.5 times nominal width, height, and depth were also analyzed, bringing the total to 20 models. As sharp transitions at strut intersections are unrealistic and cause stress singularities in a mathematical model, fillets with dimension equal to strut radii were created at interior corners of all models.

The myocardial tissue of the struts was hyperelastic, represented by a nearly-incompressible, neo-Hookean model with Lamé and bulk modulus parameters previously measured for E12.5 mouse left ventricle [13]. Models with rigid struts and those with linear elastic/large strain material properties were also analyzed. Pressure applied to one side of the cube as fully developed flow with zero pressure on the opposite side drove the fluid flow. Table 1 lists other fluid and solid parameters and Fig 3b shows boundary conditions. The model was solved in COMSOL v6.3 finite element software (COMSOL Inc., Burlington, MA) with the Multiphysics option to fully couple the laminar fluid and hyperelastic solid domains and a moving mesh in the fluid to match the solid deformation. Tetrahedral elements with order quadratic for solid and fluid velocity and linear for fluid pressure meshed the domain. A stationary, segregated solver coupled with PARDISO (PARallel DIrect SOlver) was used with relative tolerance of 0.001 for the converged solution. Specific permeability *κ* was calculated from Eq. (1), with velocity **u** represented by the average velocity across the entire cube face, equal to the area-weighted average of calculated fluid velocity for the pore area and zero for the solid area.

**Table 1:** Geometric and material parameters for the idealized unit cell, 3D explicit, 2D continuum, and 3D idealized ventricular models.

Quantity	Unit Cell	3D Explicit	2D Continuum	Idealized 3D Ventricular
Pore side (μm)	32.5, 65, 97.5, 130	65		
Pore strut radius (μm)	4 – 30	10		
Lamé constant μ (kPa)	1.94	1.94	1.94	1.94
Bulk modulus (kPa)	96.3	96.3	96.3	96.3
Fluid density (kg/m^3^)	1000	1000	1000	1000
Fluid viscosity (Pa·s)	0.0014	0.0014	0.0014	0.0014
Length (μm)		325	325	
Width (μm)		65		
Laminar zone height (μm)		65	65	
Trabecular zone height (μm)		204	195	
Compact zone height (μm)		37.5	37.5	
Porosity	0.60-0.87	0.80	0.80	0.80
Specific surface (m^-1^)	13-74			
Brinkman permeability (m^2^)			5.0-7.0 x 10^–11^	5.0 x 10^–11^
Brinkman Lamé constant μ (kPa)			0.39	0.39

### 2.4 Image-based model

The idealized single-pore analysis was repeated on an image-based model of flow at the pore scale created from the confocal microscopy scans with FIJI (an extended version of ImageJ) and the 3-D Viewer plug-in (Fig 4). After selecting five 50x50 pixel (3.6 μm/pixel) regions of interest, a stack of 50 slices (2.4 μm/slice) was imported into FIJI, converted to an 8-bit format, inverted to highlight the fluid sub-volume, and smoothed with a 3-D Gaussian Blur filter (σ = 4) to reduce excessive fine-scale features and image noise. The fluid domain was then segmented with a gray-scale threshold tool (160–170 from a possible range of 0–255) in the 3-D View plug-in and exported as an STL file suitable for importing to COMSOL v6.2. In COMSOL, boundary conditions of no-slip were applied to the wetted surfaces of the pores. Flow was induced by imposing a pressure difference from the inlet (left) to the outlet (right) surfaces with symmetry (slip) conditions applied to the lateral surfaces of the computational domain. After meshing, the image-based models were solved for the fluid pressure and velocity fields neglecting deformation of the solid domain. Other solution conditions include laminar flow, stationary solver, free tetrahedral mesh with typical element size of 10 µm in a 180-µm wide domain, and convergence at error<0.001. The total flow was integrated over the inlet to yield a value for the hydraulic permeability of the domain. The permeability was calculated for flow in each of three orthogonal directions and then averaged.

**Fig 4:**
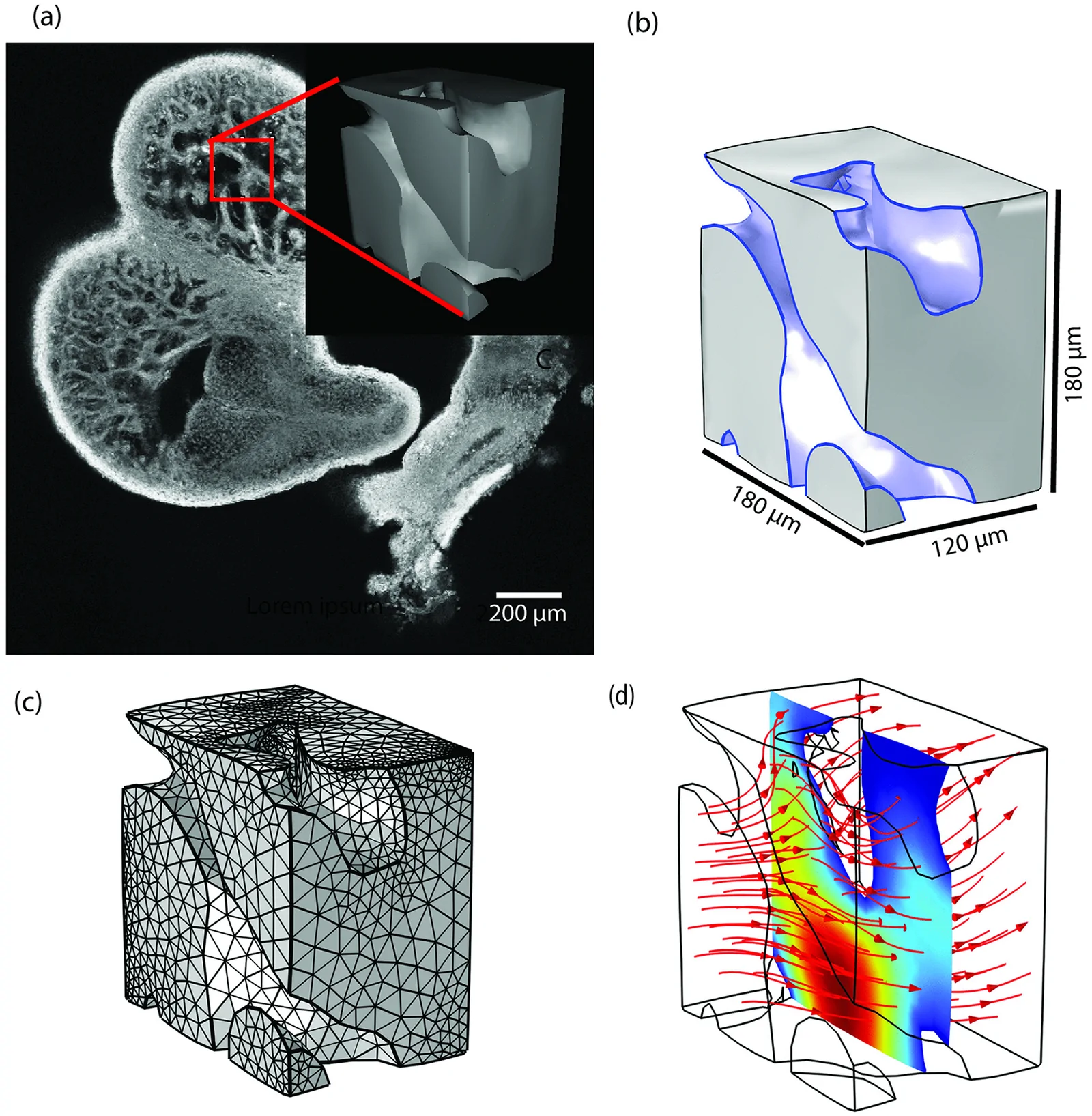
Process for creating image-based models of flow at the pore scale. (a) Selecting a region of interest followed by filtering and threshold segmentation, (b) Applying boundary conditions (inlet on the left, no-slip on wetted surfaces in blue, outlet on right rear), (c) Computational mesh, (d) Velocity magnitude on representative slices with streamlines in red.

### 2.5 Theoretical calculation of specific permeability

The computed permeabilities from both idealized and imaged-based pores can be compared to estimates based on the Navier-Stokes equations, porosity, and wetted surface area. The Kozeny-Carman equation for pore permeability of a rigid pore is [20]


$$\begin{array}{c} \kappa =\frac{\epsilon^{\text{3}}}{{Gs}^{\text{2}}}, \end{array}$$ (3)

where the specific surface is $s=\frac{A_{wetted}}{V_{total}}$ and the Kozeny constant has been reported as G=2 for circular pores and G=1.78 for square pores [16]. For fibrous media, a suggested value of κ for random, three-dimensional arrays of rigid fibers of a highly porous medium (ϵ>≈0.7)  is [21]


$$\begin{array}{c} \kappa =-\frac{\text{3}r_{f}^{\text{2}}}{\text{2}\text{0}(\text{1}-\epsilon )}\left[\text{ln}(\text{1}-\epsilon )+\text{0}.\text{9}\text{3}\text{1}\right], \end{array}$$ (4)

based on reviews of hydrodynamic calculations for arrangements of cylindrical rods and Darcy permeability data from fibrous media [22]. Here *r*_*f*_ is the fiber radius.

### 2.6 3D explicit model

The 3D explicit model represents a section of one unit-cell thickness taken from a rectilinear model (Fig 3b). The idealized structure contains the three main layers in the ventricles of the developing heart, open flow, porous trabeculated myocardium, and compact myocardium, which may also be of interest in modeling other tissues. Flow enters normal to the porous surface, allowing exploration of the effects of impinging flow, and then flows parallel to the porous interface, highlighting fluid shear effects. Three layers of the unit cell structure described in Section 2.2 represent the porous trabecular layer. Material properties and dimensions are given in Table 1 and boundary conditions in Fig 3b. Input pressure was adjusted to achieve a maximal flow velocity of 20 mm/s to match that reported in LV relaxation in early diastole in E12.5 mouse [23]. The model was solved in COMSOL v6.3 with the Multiphysics option to fully couple the laminar fluid and hyperelastic solid domains and a moving mesh in the fluid domain to match the solid deformation. Tetrahedral elements with order quadratic for solid and fluid velocity and linear for fluid pressure meshed the domain. A stationary, segregated solver coupled with PARDISO was used with relative tolerance of 0.001 for the converged solution.

### 2.7 2D continuum model

This model has the same configuration as the 3D explicit model, but replaces the explicit model of the pores with a continuum representation using Brinkman equations. Since the discrete pore structure is removed, the model can be solved in two dimensions, representing a single, two-dimensional slice perpendicular to the long axis of the structure. Material properties and dimensions of the model are given in Table 1 and boundary conditions in Fig 3c. Inlet pressure was set at 13 Pa to match that determined for the 3D explicit model. The Brinkman region permeability κ was varied from 5x10^-12^ to 5x10^-10^ m^2^ with fixed inlet pressure to examine its effect on four fluid flow variables: inlet velocity, midchannel velocity, porous flow rate, and percentage of inlet flow into the porous region. The FSI calculation in COMSOL v6.3 solved Navier-Stokes equations in the open channel, Brinkman equations in the porous region, and solid elasticity equations in the compact zone. A moving mesh in the laminar fluid domain matched the hyperelastic solid deformation. Tetrahedral elements with order quadratic for solid and fluid velocity and linear for fluid pressure meshed the domain, with a moving mesh in the fluid. A stationary, segregated solver coupled with PARDISO was used with relative tolerance of 0.001 for the converged solution.

### 2.8 Idealized 3D ventricular model

After establishing the utility of the Brinkman continuum representation of a large porous region such as exists in the developing heart, a 3D computational representation of the major features of the ventricles of an E12.5 embryonic mouse heart was created. The model contains LV and RV chambers, each with a central, open, laminar-flow region, surrounded by a porous trabecular region, in turn surrounded by a solid elastic compact wall (Fig 5). A large ventricular septal defect (VSD) connects the central LV and RV regions. The geometry is idealized, with all regions having ellipsoidal shape based on our imaging and [24], and overall dimensions representative of our measurements of the E12.5 mouse heart. The major and minor axes of the ventricular layers are 0.27 and 0.18 mm for the laminar region, 0.89 and 0.70 mm for the porous region, and 0.96 and 0.77 mm for the compact region.

**Fig 5:**
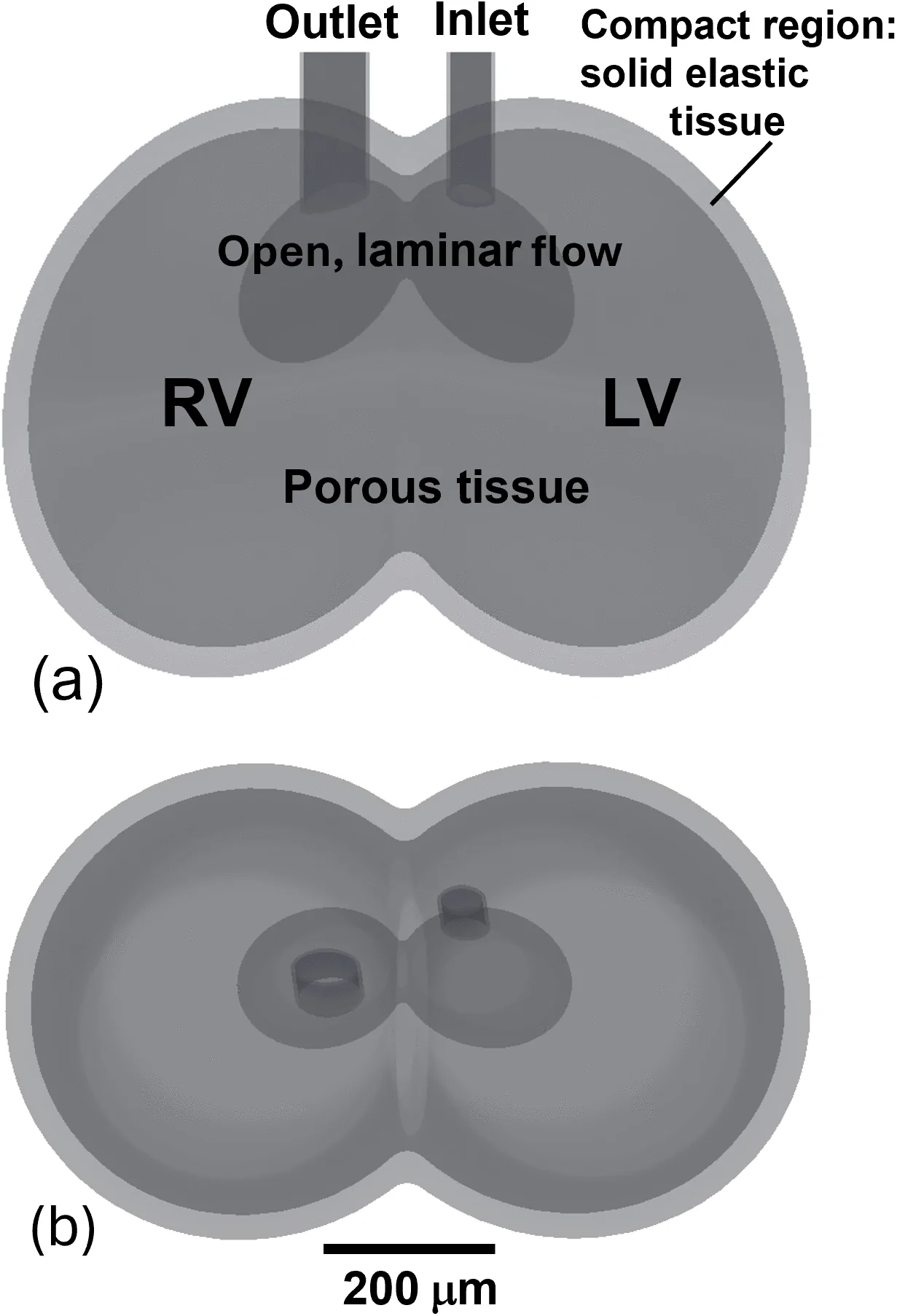
Geometry of the idealized 3D ventricular model as created in SolidWorks. (a) Midsection slice from a frontal view; (b) Top view. RV: right ventricle, LV: left ventricle.

Although the model is intended to illustrate continuum modeling of the trabecular region rather than exactly representing a specific heart, input pressure was adjusted to achieve reported intraventricular flow velocity in E12.5 mouse [23] and intraventricular pressure from developmentally matched chick [25]. To simplify the analysis of flow paths, fluid driven by constant pressure entered from a single conduit representing the primitive AV canal into the LV. Flow exited from the RV to zero pressure through a single, non-valved, non-septated conduit representing the OFT. Table 1 gives material properties used in the model for the fluid and solid. As in the 2D continuum model, the FSI was fully coupled, and the solid part of the porous region was loaded by shear stress at the laminar interface and fluid drag, in addition to the fluid pressure. The model was solved in COMSOL v6.3 with the Multiphysics option to fully couple the laminar fluid and solid domains and a moving mesh in the fluid domain to match the solid deformation. Tetrahedral elements with order quadratic for solid and fluid velocity and linear for fluid pressure meshed the domain. A stationary, segregated solver coupled with PARDISO was used with relative tolerance of 0.001 for the converged solution.

## 3 Results

### 3.1 Unit-cell and image-based model results

The specific hydraulic permeabilities for the idealized unit-cell and the image-based model were obtained from the flow calculated by COMSOL for an imposed pressure drop (Table 2). For 20 rigid models, including the rectangular cuboids, a graph of calculated permeability vs $\frac{\epsilon^{\text{3}}}{s^{\text{2}}}$ was linear with R^2^ of 0.991 and slope of 0.128, which from Eq. (3) indicates the Kozeny-Carman theory predicts the permeability well with a G of 7.82 (Fig 6). These results were independent of the applied pressure. Permeabilities of the rigid unit cells with ɛ > 0.70, and thus with relatively thinner struts, fit the rigid-fiber model of Eq. (4) with R^2^ of 0.997, but slope of 1.30 instead of 1.00. Calculated permeabilities of the rigid image-based models encompassed those of both the rigid FEM and Kozeny-Carman solutions, but the range from the five models analyzed was broad and sensitive to the image-processing parameters (Table 2).

**Table 2:** Hydraulic permeabilities calculated from finite element models (FEM) and estimated from the Kozeny-Carman (K-C) equation using G = 7.82 for image-based pore-scale models. The range is from the means of three orthogonal directions at five different locations within the image.

	3D Image-Based	
Segmentation Threshold (0–255)	160 (N = 5)	170 (N = 5)
ε	0.56-0.73	0.44-0.57
s (m^-1^)	1.03- 2.06 x 10^4^	1.62 - 2.01 x10^4^
κ (m^2^) FEM (rigid)	0.690 - 6.33 x 10^–10^	0.26 – 1.22 x 10^–10^
κ (m^2^) K-C, G = 7.82 (rigid)	0.43 -10.3 x 10^–9^	1.41 – 7.27 x 10^–10^

**Fig 6:**
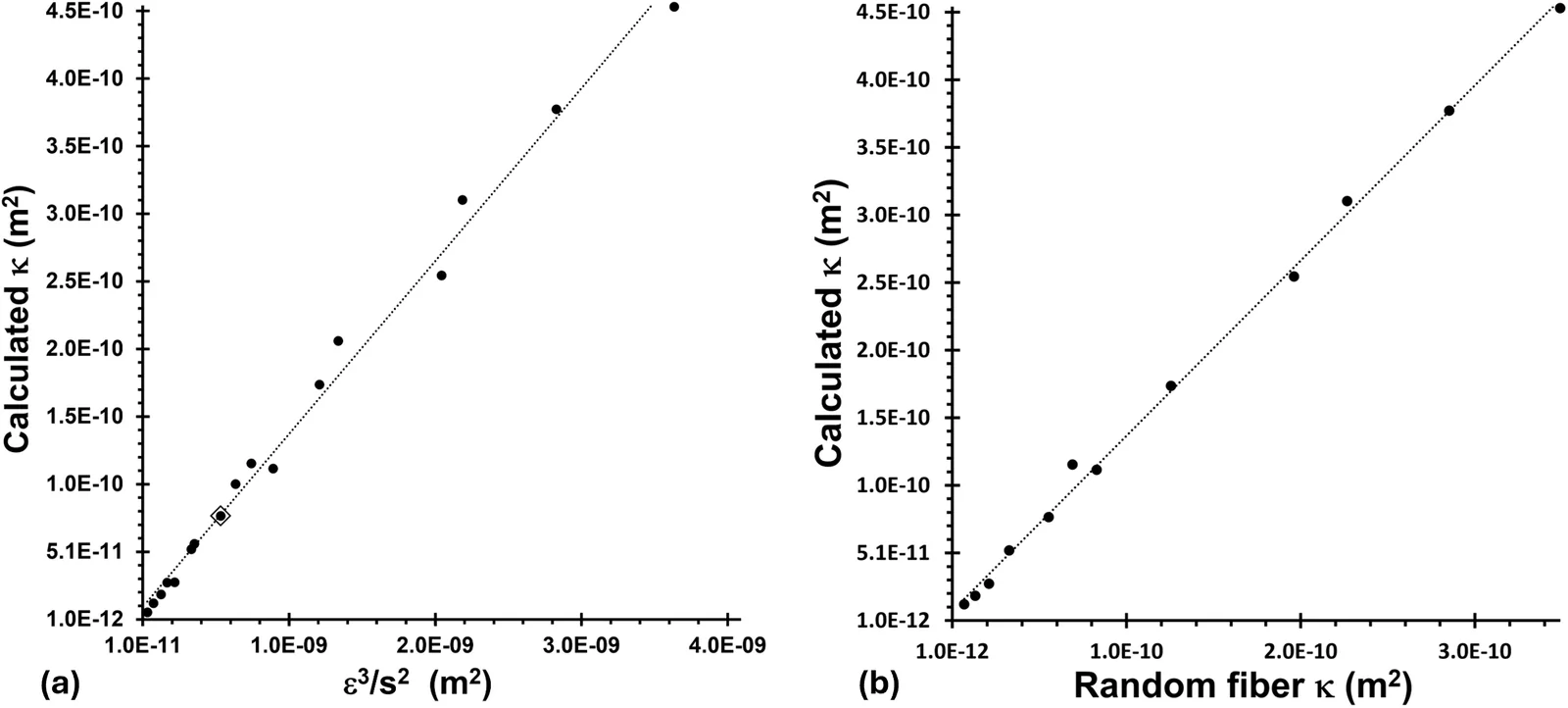
Calculated rigid unit-cell specific hydraulic permeability κ compared to Kozeny-Carman models. (a) Permeability calculated for 20 models of varying cube side and strut radius plotted versus the Kozeny-Carman theory of Eq. (3). Open diamond represents rectangular cuboid shapes. Dotted line shows best linear fit with R^2^ of 0.991 and slope of 0.128. (b) Calculated permeability of unit cells with porosity greater than 0.70 plotted versus the theory for random fiber arrays of Eq. (4). Dotted line shows best linear fit with R^2^ of 0.997 and slope of 1.30.

Calculated permeabilities of the elastic unit cells were lower than those of the corresponding rigid models, the difference increasing quadratically with pressure across the pore and the effect more pronounced on more flexible struts. For the nominal pore geometry, permeability decreased by 7% at 10 Pa and 16% at 15 Pa over the rigid case, while for the same cube side with 20% thinner struts, permeability decreased by 18% at 5 Pa. Analyses of models with linear elastic properties showed that permeability was only weakly dependent on elastic modulus, increasing by 10% for a 5-fold increase in E.

Fluid velocity increased somewhat slower than linearly with pore pressure difference, due to the compression of the fluid channels between the struts as the deformation increased (Fig 7a). For example, with the nominal geometry, the maximum velocity was 18.2 at 5 Pa and 33.7 at 10 Pa. Fluid paths compressed when passing between struts and expanded in between (Fig 7a). Solid stress and strain were largest at the junction of the trailing vertical and horizontal struts. First principal stress maxima at the trailing strut lower corners was 100 times pore pressure difference (Fig 7b). The main mode of pore deformation was sliding of the top surface in the flow direction (Fig 7b). WSS was largest on the struts bordering the inlet and outlet regions, approximately symmetric on the surfaces lateral to the flow, and largest in the centers of the struts (Fig 7c). Maximum WSS for the nominal pore increased with pressure difference, being 0.88 Pa at 1 Pa pressure, 4.7 Pa at 5 Pa, and 12.2 Pa at 10 Pa.

**Fig 7:**
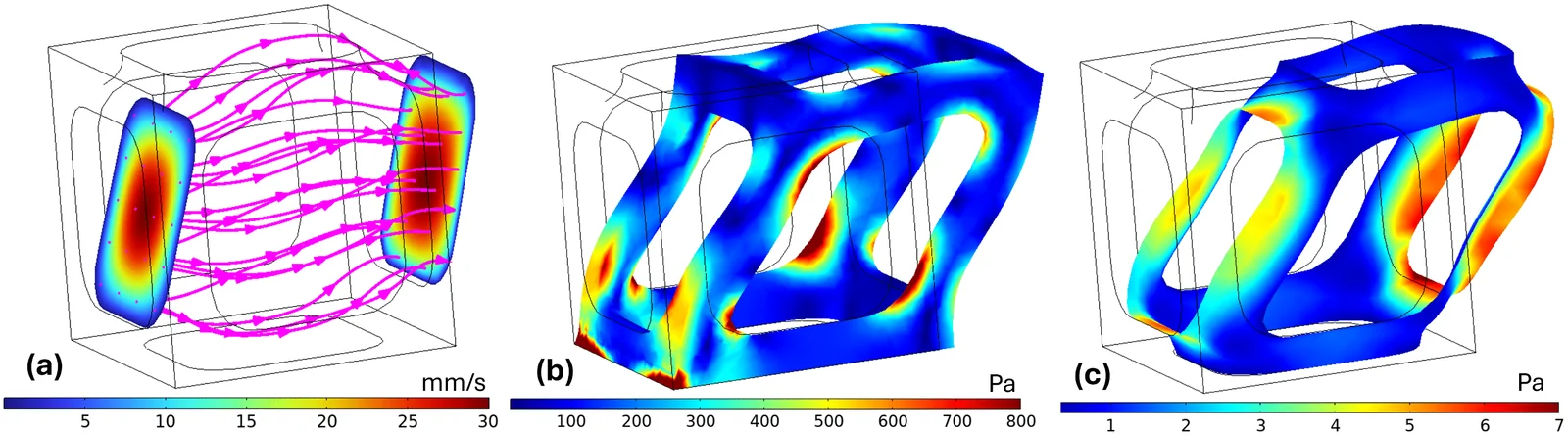
Results from the idealized unit-cell model. Cube side is 65 μm, strut and fillet radii are 10 μm, and inlet pressure is 10 Pa. Deformation is shown at 1:1 scale, and gray outline shows undeformed geometry. (a) Fluid velocity magnitude and streamlines showing maximal velocity in the center of cross-sections and flow expansion and contraction at inlet and outlet. Arrows show direction of flow. (b) Von Mises stress with maximum of 1 kPa at the trailing inner fillets. The corresponding strain maxima are 0.20 at the trailing inner fillet and -0.16 at the leading inner fillet. (c) Wall shear stress with maximum of 12 Pa on the trailing inner edges of the struts.

### 3.2 3D explicit and 2D continuum model results

In the 3D explicit model, pore pressure difference was adjusted to 13 Pa to produce the 20.4 mm/s blood velocity measured in LV relaxation in early diastole in E12.5 mouse [23]. The inlet flow rate was 0.058 mm^3^/s while flow into the porous region was 0.0105 mm^3^/s, so most entering fluid, 82%, flowed through the open channel, never crossing into the porous region (Fig 8a, 8c).Fluid velocity in the porous region was small, with local increases when flowing between struts, as can be seen in the compressed velocity scale of Fig 8c. Fluid entered each pore level through the first two pores only, and exited mainly through the last two pores, with only 1% exiting through the middle pore. Of the 18% of inlet flow entering the porous region, 49% flowed through the first pore layer underneath the open-channel region, with 30% flowing through the 2^nd^ layer and 21% through the 3^rd^ layer.

**Fig 8:**
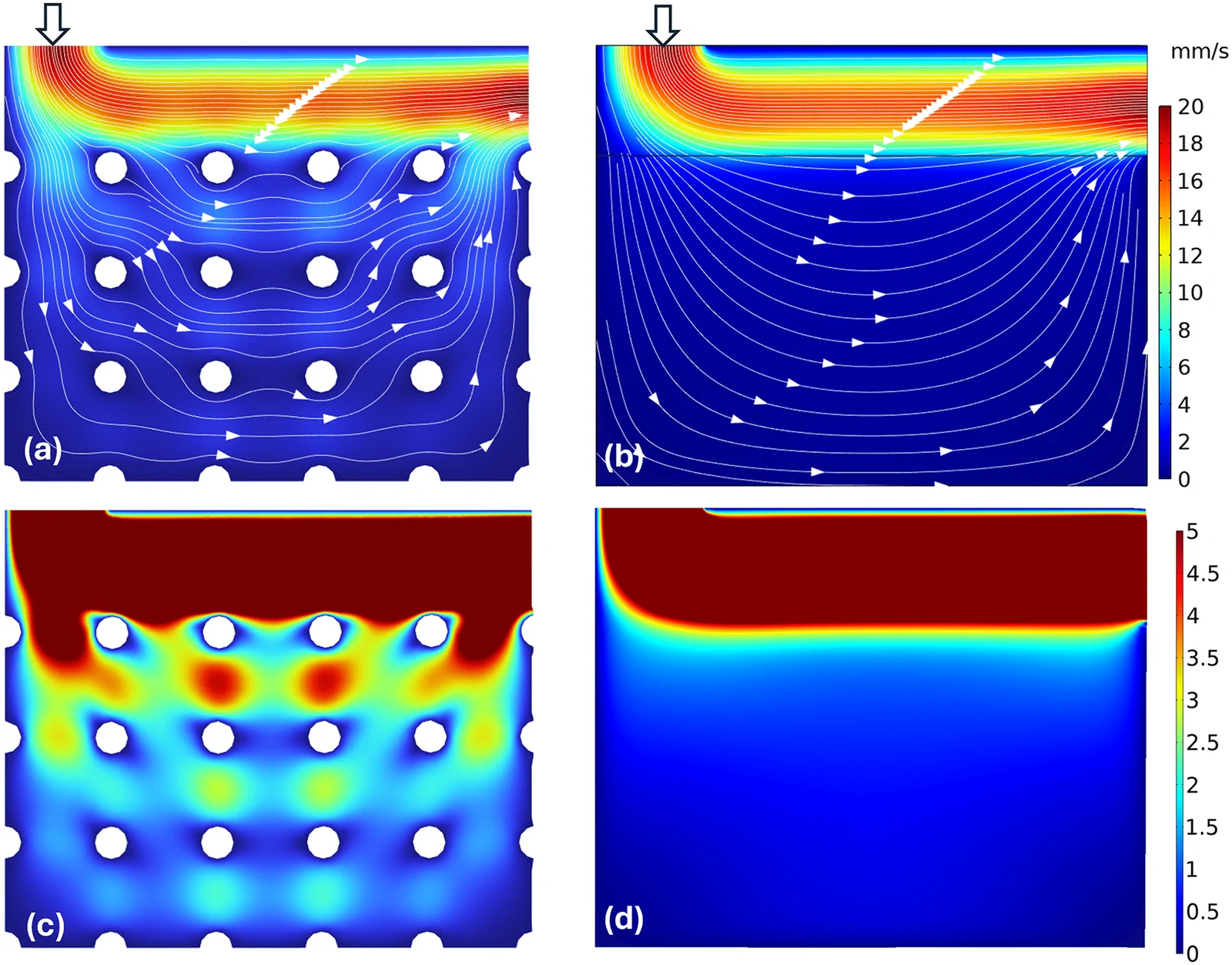
Comparison of fluid velocity in the 3D explicit and 2D continuum models. (a) Midplane of explicit model with 13 Pa driving pressure. Streamlines are shown in white; large arrow indicates location of fluid inflow. (b) Continuum model with κ = 5x10^-11^ m^2^ and equivalent inflow. Streamlines are shown in white; large arrow indicates location of fluid inflow. The color scale applies to both (a) and (b). (c) Midplane of explicit model with a compressed velocity range to better show flow velocity between struts. (d) Continuum model with the same compressed range. The color scale applies to both (c) and (d).

In the 2D continuum model, most entering fluid also remained in the open channel (Fig 8b, 8d), and the velocity distribution in the open channel was similar to that in the 3D explicit model. Fluid velocity in the porous region was largest closest to the porous-open interface and decreased continuously with depth in the porous region, without the acceleration between pore struts seen in the explicit model. Fluid velocity at the interface with the compact region was approximately equal in both models. As in the explicit model, fluid entered the porous region in the first half of the length, and exited in the second half of the length. With a Brinkman region permeability of κ = 7x10^‑11^ m^2^ as calculated for the nominal idealized pore, maximum velocity was 22.9 mm/s and 23% of the inlet flow entered the porous region. Mid-channel velocity was within 0.70% of the explicit model solution. Reducing the permeability to κ = 5x10^-11^ m^2^ gave the best match to target flow parameters of the explicit model (inlet and mid-channel velocity, porous flow rate and percentage of inlet flow) with inlet velocity of 20.4 mm/s, flow rate into the porous region of 0.0107 mm^3^/s, and percentage of inlet flow into the porous region of 18.4%. Changing permeability by a factor of 10, to 5x10^-12^ or 5x10^-10^ m^2^, resulted in only modest changes in flow parameters, -24% and 43% in inlet velocity and flow, ± 05% in mid-channel flow, and ±85% in porous flow.

Fluid pressure was similar in magnitude and distribution in the explicit and continuum models, decreasing in the direction from inlet to outlet and with depth into the tissue (Fig 9a, 9b). Despite the similarity of pressure, calculated solid stress and shear stress differed between the two models, which have dissimilar load-carrying sections exposed to flow. In the explicit model, solid stress was largest in the struts adjacent to the fluid inlet, with maximum von Mises stress of 150 Pa in the first pore fillet, and was generally larger everywhere at fillets and low in the compact zone (Fig 9c). In the continuum model, solid stress was largest in the compact zone with a maximum of 14 Pa at the junction with the porous region (Fig 9d). In the explicit model, fluid shear stress was largest on the rounded tops of struts directly adjacent to the open channel, where fluid must flow around these barriers, and on the walls of the channel (Fig 10a, 10b). The peaks of shear stress seen on the tops of the struts in the explicit model were absent in the continuum model (Fig 10c).

**Fig 9:**
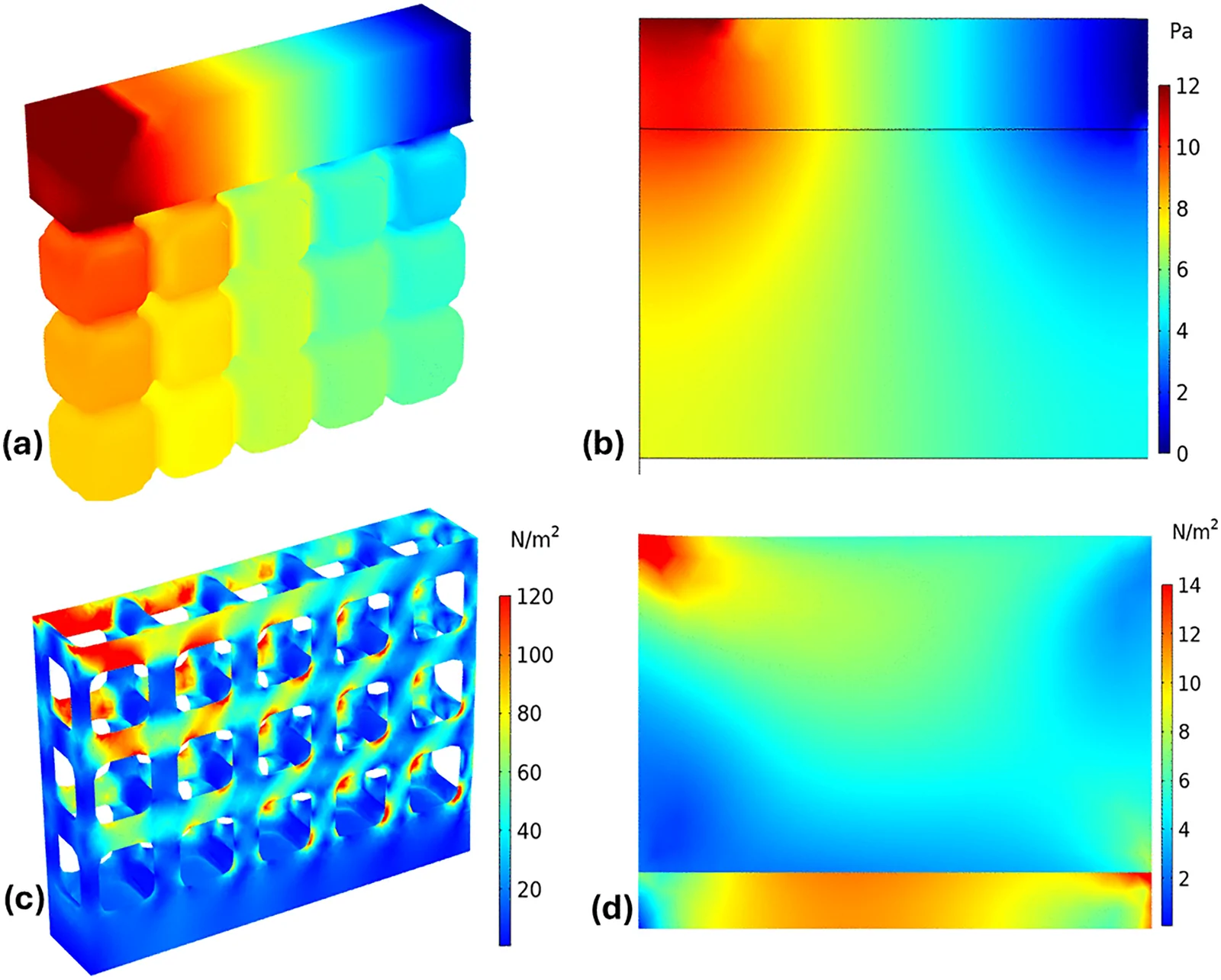
Fluid pressure and solid stress in the explicit and continuum models. (a) Fluid pressure in the open channel and porous regions of the 3D explicit model with 13 Pa inlet pressure; (b) Fluid pressure in the open channel and porous (Brinkman) regions of the 2D continuum model κ = 5x10^-11^ m^2^. The scale at right applies to both (a) and (b). (c) Von Mises stress in the 3D explicit and (d) 2D continuum model. The peak magnitudes were more than eight times larger in the explicit model and a reduction in color scale range was necessary in (d), as the figure would be uniformly blue with the same color scale as (c).

**Fig 10:**
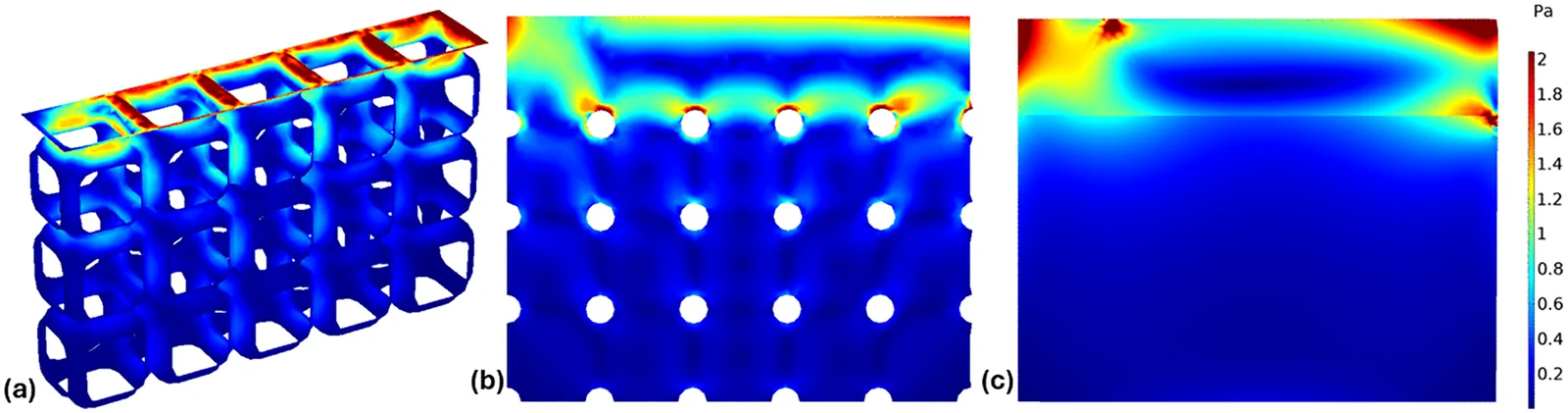
Calculated wall and fluid shear stresses. (a) Explicit model, (b) Midplane of the explicit model, and (c) Continuum model. The color scale of stress in Pa at right applies to all three panels.

### 3.3 Idealized 3D ventricular model

Inlet pressure was adjusted to 1 kPa to replicate physiological targets for intraventricular velocity [23] and pressure [24]. Fluid entered the LV at a velocity of 250 mm/s and decreased to 20 mm/s before flowing through the VSD. Pressure decreased rapidly nearing the entrance to the LV and was approximately uniform at 208 Pa within the laminar and porous regions The inlet flow rate was 0.15 mm^3^/s, while the total outward flow rate from the open cavities into the porous region was 0.073 mm^3^/s, or 47.7% (Fig 11a). Most fluid flow out of the open central cavities occurred just before the sulcus between the ventricular cavities and near the outlet, while most flow into the cavities occurred just after the sulcus and near the inlet (Fig 11b). At the laminar-porous interface, WSS was up to 2 Pa on surfaces just after the inlet, 0.45 Pa in bands around the VSD (Fig 11c). The first principal stress was highest in the sulcus, up to 900 Pa, with otherwise broad regions of 350–400 around the outside edge and 400–450 around the bottom edge (Fig 11d). Trials varying the size of the open cavity and VSD showed that the magnitude of stress was sensitive to these features, although the general patterns did not change.

**Fig 11:**
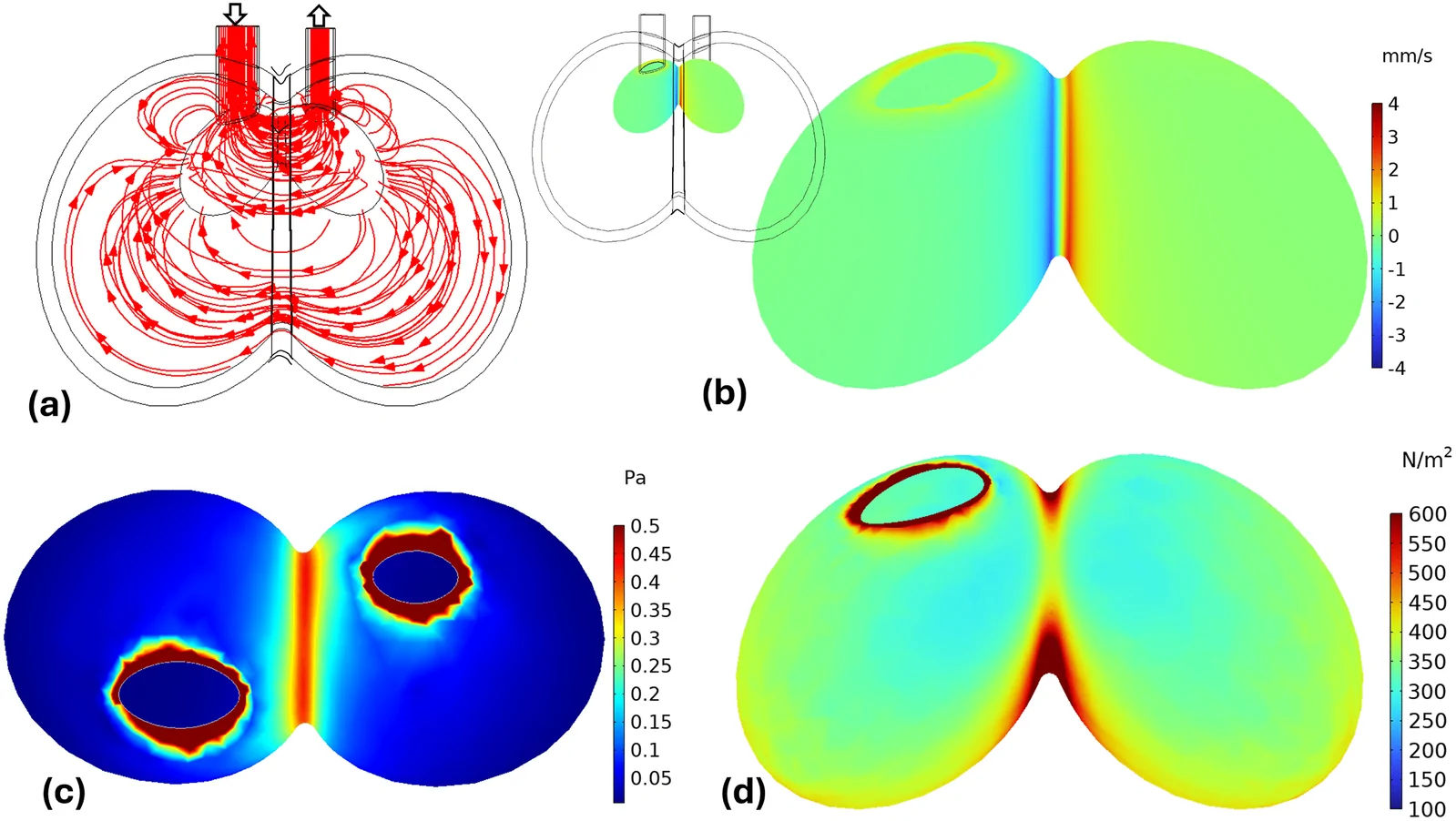
Fluid velocity, solid stress, and wall shear stress in the ventricular model. (a) Streamlines of flow in the idealized ventricular model, shown on a frontal midplane slice, with arrows showing the direction of fluid inflow and outflow. Figs (b-d) show only the interface surface between the open central cavities and the porous region. (b) Fluid velocity normal to the surface seen from the front. Relatively little flow occurs in regions far from the inlet, outlet, and interventricular connection. Positive velocities (yellow-red colors) indicate flow out from the porous region, while negative velocities (blue colors) indicate flow into the cavities. Inset figure shows the location of the surface within the entire model. (c) Wall shear stress as seen from the top. (d) First principal stress viewed from the front.

## 4 Discussion

This work used three levels of models to develop and verify the utility of a computationally efficient continuum representation of poroelastic materials and its application to studies of the trabeculated embryonic heart. Characterization of the poroelastic material requires three parameters: porosity, elasticity, and specific hydraulic permeability. Permeability was relatively insensitive to elasticity within a reasonable range, and porosity can be estimated from imaging studies. Permeability, however, is crucial and can be impossible to obtain experimentally, given the small amount of tissue available and its fragility in the case of the embryonic heart.

Trials with various sizes of rigid unit-cell models, perturbations of the nominal pore measured from confocal microscopy of E12.5 mouse ventricles, showed that the prevailing Kozeny-Carman solid-pore or fiber theory fit the trend well and provided a fitting constant G. This is useful as a first estimate of permeability when accounting for variable size pores. The difference in G compared to other published values (e.g.[16],) may be due to the non-uniform cross-section of our models, where the fluid path must expand after passing the struts surrounding the inlet and contract through the struts surrounding the outlet (Fig 7a). This effect more than counteracts what would otherwise be an increase in permeability of 15–20% over theory due to the slip boundary condition on the side walls representing interfaces with adjoining pores. The models are useful to quantify the effect of elasticity, which reduces permeability as some flow energy is converted into material deformation and the flow area is reduced. We note that all of the permeabilities estimated in this study of the developing heart wall are orders of magnitude higher than those experimentally measured, such as 1 ‑ 6 x10^-15^ in the abdominal wall [26], 0.3 - 5 x10^‑18^ in the aorta [27–29], 0.9 - 23 x10^-15^ in various tumors [30], and 1–6 x10^-14^ in extracellular matrix [31], all in m^2^ units.

While rigid image-based models were successfully created from several representative regions of the image stacks, meshing was not always feasible. The filter and thresholding parameters that yielded usable models tended to overestimate the strut thickness and underestimate the porosity. As a result, we found that the Kozeny equation could predict the permeability reasonably well from porosity and wetted surface for the image-based models that could be meshed, but the values of the porosity differed somewhat from those used for the idealized geometry models. The parameters used to smooth and threshold the image affect the estimation of the permeability primarily by altering the estimated thickness of the struts as reflected in the differences in porosity and surface area to volume ratios. The process of smoothing the images enough to allow meshing also loses some detail in the cross-section of the struts, which can cause our methods to underestimate the wetted surface area and fluid volume. A large body of literature exists on the sensitivity of permeability and stiffness to the unit cell geometry and dimensions [32–43]. Nonetheless, the image-based models served as useful validation of our pore-scale models based on idealized geometries and illustrates some of the potential challenges in image-based modeling.

The Brinkman region permeability in the continuum model that produced the best match of flow objectives (inlet and mid-channel velocity, porous flow rate and percentage of inlet flow) from the 3D explicit model was only slightly reduced from that calculated from the nominal unit-cell model. The particular geometry chosen for the explicit model, the location of the inlet, and the use of inlet pressure as fixed may influence this. Additionally, the geometry of the strut tops bordering the open channel affects the apparent permeability of the porous layer. A 3D explicit model with flattened tops bordering the open channel reduced the difference in flow objectives by more than half. Also, the reduction in best-match permeability is small compared to the range of permeabilities calculated for the various unit-cell geometries and for biological tissues in general. However, since permeability is the most important determinant in a continuum model, and the methods used to estimate the bulk properties of a porous region can yield widely different values, it emphasizes the need to perform parametric studies that account for the likelihood that the effective permeability properties used in a whole organ may vary by an order of magnitude or more, just as details of the microstructure may vary among individuals.

The models created here incorporated several assumptions. Measurements of blood viscosity and density for E12.5 mouse are not available in the literature and were therefore estimated from developmentally matched chick. The assumed blood dynamic viscosity μ of 0.0014 Pa•s matches that of HH22 (E3.75) chick at a shear rate of 100 s^-1^ [44]. For comparison, μ in adult wild-type mouse is 0.003 Pa•s [45] and that of water at 37^o^C is 0.00069 Pa•s. A blood density ρ of 1000 kg/m^3^ was assumed [46] because of the low hematocrit of 0.194 measured in HH22 chick [44]. Permeability was assumed as isotropic, but anisotropy can be easily incorporated in software if the permeability tensor is known. The methods introduced here are based on steady-state analyses of pressure, flow, and stress. Pulsatile flows also behave as quasi-steady state when the flow rate keeps up with the instantaneous pressure gradient and transient fluid inertial forces can thus be ignored. The significance of possible transient inertial effects in time-varying flows is typically determined by estimating the Womersley number α [47–49] defined as


$$\begin{array}{c} \alpha =L{\left(\frac{\omega \rho }{\mu }\right)}^{\text{1}/\text{2}}\;. \end{array}$$ (5)

Here L is a length scale and ω is the radian frequency of time variation. When α < 1, velocity profiles, shear stresses, and pressure drops are expected to show little difference due to inertial effects. For the example of the E12.5 mouse ventricles, a typical heart rate is 150 BPM [24]. For the unit cell, using ρ = 1000 kg/m^3^, μ = 0.0014 Pa ∙ s, and L = 65 μm gives α = 0.22, indicating that inertial effects are small and changes in velocity can be handled quasi-statically. Even at the larger scale of a ventricular chamber (L = 200 μm), inertial effects remain small. We therefore expect this model to be valid when pulsatile flow is imposed. A similar check of the Womersley number should be made for applications to other time-varying situations and anatomy.

Experimental measurements are important for model verification, but assumptions and manipulation are necessary to obtain these in an organ as fragile as the early embryonic heart. For example, in the more easily accessible chick with solid tubular geometry, WSS calculation in the proximal OFT at HH23 (4d) required *ex ovo* embryo culture and approximation of the flow field in boundary layers [50]. In slightly older chick (HH 25), egg windowing was necessary for collection of Doppler data used in CFD calculation of WSS in the OFT [51], while wall motion estimation and omission of individual trabecular geometry extended this to the ventricle [52]. Mouse models, important in allowing genetic manipulations that mimic human congenital heart disease [15,53,54], present greater challenges to *in vivo* blood flow and pressure measurements. As demonstrated in a study assessing diastolic function in E9.5-19.5 mouse, obtaining limited apical Doppler velocity and single-point pressure probe measurements in the ventricle required an anesthetized dam, removal and opening of part of the uterus, limited time, and inability to obtain all hemodynamic information on the same embryo [23]. Thus, robust verification of quantities such as pressure drop across small distances or mapping of flow velocity over an intraventricular border is difficult, highlighting the value of multiple parameter sweeps and boundary condition variation to match limited physical measurements.

The idealized ventricular model does not explicitly include myocardial contraction. Flow simultaneously enters and exits the ventricles, encompassing features of both systole and diastole. These phases, which include an A wave from atrial contraction in late diastole propelling blood into the ventricle and ventricular isovolumic contraction and relaxation, already exist in the E12.5 mouse heart, even without mature valve tissue [55]. Myocardial contraction would involve squeeze flow from deeper pore levels, which should be mostly radial, as symmetry eliminates most circumferential flow. The velocity of the blood moving past the struts of the porous layer is then approximately the speed of radial contraction, which can provide an estimate of WSS. Full active models are beyond the scope of this research, but can be facilitated with the techniques developed here.

This study is unique in including fluid flow and FSI in a model of the trabeculated developing heart. Previous models of the embryonic heart have considered formation of trabeculae in a flat sheet of cardiac jelly with embedded contractile fibers [56] static solid mechanics of a cylindrical HH21 chick model with porous inner layer under internal pressure [57], and a multiscale derivation of a strain energy density function for the trabecular region of HH21 chick [58], later used in a finite element model calculating strain patterns in the looped heart [59]. Explicit 3D trabecular geometry from HH26 (E6.0) chick was used in one study of solid stress under static internal pressure [12]. Cairelli et al. [60] studied WSS in 3-dpf zebrafish hearts, which have a much simpler trabecular structure: a single ventricle with less than 10 trabeculae in a corrugated appearance around the outer wall. From reconstruction of confocal images and image-based CFD, the study found that a squeeze flow effect was responsible for most of the WSS magnitude in the intra-trabecular spaces, rather than the shear interaction with the flow in the main ventricular chamber.

Comparison of the 3D explicit and 2D continuum models showed that a large region of regular pores can be represented as a porous continuum and, although fluid velocity decreases with depth, the Brinkman model is necessary to account for fluid shear effects near the interface between the open channel and the porous trabecular wall. Although predictions of volumetric flow rate and pressure from the Brinkman model closely matched the 3D explicit model, the localized peaks of WSS and solid stress were absent, such as the velocity gradient between pores, local peaks of WSS on strut tops, and corner stress in the unit cell struts. Post-processing estimators are thus necessary to use continuum results from the whole organ to estimate the experience of cells in a porous region. The prediction of pressure from the continuum model can be used with local explicit geometry and boundary conditions to estimate solid stress. A number of possibilities exist for WSS estimation.

The 3D explicit model showed that high WSS is almost exclusively restricted to the surface layer of the porous region exposed to the open-channel flow (Fig 10a, 10b). One method of estimating WSS from continuum results involves modeling flow past a cylinder in an infinite domain with a free stream velocity equal to the maximum velocity in the open channel. Theoretical solutions for low-Reynolds flow exist, with the shear stress on the tops of the semicircular struts given by $\tau_{r\theta }=\frac{C\mu u_{\infty }}{R}$, the value of C dependent on the Reynolds number [61]. We modeled a long channel with no-slip condition on walls and a transverse cylinder in the middle and found that shear stress varies inversely with channel height except for the narrowest channels, with height less than three times cylinder diameter. Using variations of channel height, velocity, and strut radius confirmed the functional dependence of WSS on h, R, u_∞_, and μ such that $\tau_{r\theta }\propto \frac{\mu u_{\infty }}{hR}$.

Another method of estimating WSS from continuum results involves force equilibrium. This can be seen by comparing forces at the porous boundary of the 2D continuum and 3D explicit models. Integrating WSS there in the continuum model over the length gives a total force of 1.66x10^‑8^ N, within 10% of the 1.84x10^-8^ N found by integrating over the strut tops facing the open channel in the explicit model. In a 3D explicit model with flattened strut tops facing the open channel, the total shear force on the strut tops was 1.64x10^‑8^ N, within 1.3% of the total force in the 2D continuum model. Thus, shear stress can be estimated by dividing the shear force in a continuum region by the relevant area.

If an estimation of WSS within pores is desired from continuum results, a possible model is flow through a noncircular duct. The maximum shear stress in the duct is $\tau_{max}=C_{f}\rho \frac{{\overset{―}{u}}^{\text{2}}}{\text{2}}$, where $C_{f}$ is a coefficient of friction and $\overset{―}{u}$ is the mean velocity over the pore opening. The value of $C_{f}$ depends on the Reynolds number in the pore, where $Re_{D_{h}}=\frac{\rho D_{h}\overset{―}{u}}{\mu }$ with the hydraulic diameter given by $D_{h}=\frac{{\text{4}A}_{pore}}{P}$, where *A*_*pore*_ is the cross-sectional area of the pore and *P* is the perimeter of pore opening. For a square pore opening, $C_{f}=\frac{\text{1}\text{4}.\text{2}}{Re}$ for Re < 10 [62]. For the rigid, single-pore model, this equation provides estimates of WSS that are within 17% of that averaged across a strut at exit for the nominal model, consistent across a wide range of pressures.

In conclusion, the process detailed here shows a computational framework for CFD modeling of a large porous region, demonstrating the importance of specific hydraulic permeability and the use of a Brinkman porous flow model to account for fluid shear stress effects near an open channel. Such organ-level models are efficient compared to computationally expensive explicit pore modeling and allow parametric analyses, while providing good solutions for fluid pressure, flow in open regions, and average flow into and within porous regions. Continuum models can then be the basis for either more localized explicit models or for post-processing estimations of localized stress, such as the force balance, flow past cylinder, and duct flow methods discussed here. In application to the developing heart in the chamber formation and OFT septation stages, they can help elucidate dependencies important in mechanotransduction, thus supplementing and better focusing *in vivo* measurements of flow or strain.
